# Unique features of a recombinant haemagglutinin influenza vaccine that influence vaccine performance

**DOI:** 10.1038/s41541-021-00403-7

**Published:** 2021-12-02

**Authors:** Arun B. Arunachalam, Penny Post, Deborah Rudin

**Affiliations:** 1 Analytical Sciences, R&D Sanofi Pasteur, 1 Discovery Drive, Swiftwater, PA 18370 USA; 2 Regulatory Affairs, Protein Sciences, a Sanofi Company, 1000 Research Parkway, Meriden, CT 06450 USA; 3 Global Medical Affairs, Sanofi Pasteur, 1 Discovery Drive, Swiftwater, PA 18370 USA

**Keywords:** Vaccines, Protein vaccines

## Abstract

The influenza vaccine field has been constantly evolving to improve the speed, scalability, and flexibility of manufacturing, and to improve the breadth and longevity of the protective immune response across age groups, giving rise to an array of next generation vaccines in development. Among these, the recombinant influenza vaccine tetravalent (RIV4), using a baculovirus expression vector system to express recombinant haemagglutinin (rHA) in insect cells, is the only one to have reached the market and has been studied extensively. We describe how the unique structural features of rHA in RIV4 improve protective immune responses compared to conventional influenza vaccines made from propagated influenza virus. In addition to the sequence integrity, characteristic of recombinant proteins, unique post-translational processing of the rHA in insect cells instills favourable tertiary and quaternary structural features. The absence of protease-driven cleavage and addition of simple N-linked glycans help to preserve and expose certain conserved epitopes on HA molecules, which are likely responsible for the high levels of broadly cross-reactive and protective antibodies with rare specificities observed with RIV4. Furthermore, the presence of uniform compact HA oligomers and absence of egg proteins, viral RNA or process impurities, typically found in conventional vaccines, are expected to eliminate potential adverse reactions to these components in susceptible individuals with the use of RIV4. These distinct structural features and purity of the recombinant HA vaccine thus provide a number of benefits in vaccine performance which can be extended to other viral targets, such as for COVID-19.

## Introduction

Seasonal influenza is responsible for 290,000–650,000 deaths per year due to respiratory diseases alone and 3–5 million cases of severe illness worldwide^1–3^. In the USA, influenza was thought to account for 52.7 hospitalisations per 100,000 people during the 2019–2020 season. These numbers are significantly higher in young children and adults aged 65 and older^1,2,4^. Extra-pulmonary complications of influenza infection constitute a further under-recognised disease burden^4,5^. Overall, such a high disease burden carries substantial social and economic cost^6,7^. Prevention of seasonal influenza epidemics, as well as preparedness for future pandemics, is thus a global priority.

Influenza A subtypes H1N1 and H3N2, and influenza B lineages B/Yamagata and B/Victoria circulate routinely in humans and are included in seasonal influenza vaccines^8^. Influenza A subtypes can also give rise to highly pathogenic viruses through cross-over from animal reservoirs to humans^9^. In the past century, four novel influenza A virus strains have emerged in this way, each leading to a global pandemic (H1N1 in 1918; H2N2 in 1957; H3N2 in 1968; and H1N1 in 2009). Vaccines against such strains are prepared and stockpiled as government initiatives for emergency use in potential future pandemics.

Haemagglutinin (HA) is the primary antigen in the induction of a protective immune response against the influenza virus, and thus a key vaccine target. Expressed as trimeric glycoproteins on the viral surface, HA binds to sialic acid on target cells to facilitate host cell entry and mediates the fusion of the viral envelope to the late endosomal membrane. Neutralising antibodies that block HA effectively prevent viral entry into target cells and have been shown to protect the host from infection^10,11^. Another viral surface protein, neuraminidase (NA), cleaves sialic acid and releases budding virus from the infected cells, thus serving as another important vaccine target. Although the presence of NA is not required for effective vaccine performance^12^, its inclusion in annual influenza vaccination may help to broaden protection and reduce influenza severity^13,14^.

The preparation of the annual influenza vaccine firstly requires the identification of the influenza strains and their like strains, most likely to spread during the upcoming season, for inclusion in the vaccine. Identification of the target influenza strains is based on surveillance data collected by World Health Organization (WHO) collaborating centres at six locations in the UK, USA (including the Centers for Disease Control and Prevention [CDC]), Japan, China and Australia as part of the WHO Global Influenza Surveillance and Response System (GISRS)^15^. The final decision on resulting vaccine targets is made by individual regulatory bodies.

Influenza vaccines can afford significant protection against influenza illness, even when there is an antigenic mismatch against the predominant circulating virus strains^16,17^. Such cross-protection can occur through the priming of the immune system by vaccination or natural infection and is primarily due to antibodies specific to conserved regions on the HA head and stem^18^. Vaccines that can induce immunity specific to circulating wild-type strains and cross-protection to related strains would be ideal against constantly evolving influenza viruses. As such, the conserved regions on the HA head and stem present attractive targets for development of universal influenza vaccines^19,20^.

The annual production of influenza vaccine through conventional, mostly egg-based platforms, is arduous and a race-against-time^21^. The production process, from the selection of influenza strains to vaccine manufacture and release for distribution, takes eight to nine months each year. Agile, reliable and egg-free technologies allowing for guaranteed and faster manufacture of influenza vaccines are needed to ensure timely delivery for upcoming epidemic seasons and during potential avian flu pandemics, when egg supply may be impacted. The adoption of alternative vaccine development approaches (including mRNA, vector, and recombinant protein strategies) in the urgent response to the COVID-19 pandemic has demonstrated the feasibility of using more efficient methods to produce new, effective vaccines within accelerated development timelines. The recombinant quadrivalent influenza vaccine (RIV4, Flublok^®^, Supemtek^®^ [EU, Canada], Sanofi Pasteur) was the first licensed influenza vaccine to be produced using recombinant viral proteins instead of antigens derived from live influenza virus (as for inactivated split-virion and subunit vaccines). RIV4 is an unadjuvanted vaccine containing 45 µg of HA/dose from each of the four strains. The production of RIV4 is based on a novel production platform in which recombinant HA (rHA) is expressed in insect cells using a baculovirus expression vector system (BEVS)^22^. In brief, *expres*SF+ insect cells are infected with recombinant baculovirus carrying the relevant influenza HA genes, which are expressed under the control of a baculovirus polyhedrin promotor. rHA molecules from the infected cells are extracted using detergent and purified from the clarified cell extract using column chromatographies followed by Q membrane filtration. Purified rHA is suspended in relevant buffer using tangential flow filtration and passed through sterile filtration for storage and formulation^22^. RIV4 has undergone extensive clinical assessment^23^, and was first approved by FDA in 2013. It is now licensed in the USA, Canada, Europe, Australia, and various other countries.

This review focuses on the structural features of BEVS-derived rHA that make RIV4 unique from conventional vaccines, and how these features help to maximise vaccine performance. Notably, the benefits of this manufacturing process can be extended to other viral targets, such as COVID-19, where the preservation of conserved epitopes is critical for imparting cross-protection against a constantly evolving and mutating virus.

## Potential antigenic mismatch of influenza vaccine virus grown in egg or cells due to adaptive mutations in the HA primary structure

A known risk of traditional split or subunit vaccines is the potential for the candidate vaccine virus or working virus seeds to acquire adaptive mutations as they grow in embryonated chicken egg or mammalian host cells during vaccine manufacture. Such adaptive mutations in HA peptides may reduce the effectiveness of the resultant vaccine^24–26^. Raymond et al.^24^ showed that an egg-adapted A/California/07/2009 (H1N1) vaccine strain acquired a mutation resulting in the substitution of glutamine with arginine at position 226 which in turn induced antibodies specific to receptor binding site that bound to vaccine-derived HA preferentially over the circulating wild-type virus^24^. During the 2012–2013 northern hemisphere influenza vaccination campaign, HA from an egg-adapted A/Victoria/361/2011 (H3N2) virus used for vaccine manufacturing differed from the WHO-recommended prototype and several other wild-type influenza viruses in three positions, H156Q, G186V, and S219Y^25^. The low vaccine effectiveness (41%) observed for H3N2 in the 2012–2013 season was attributed to these three mutations during vaccine production^25^. Other antigenic mutations introduced by egg-adaptation of the vaccine strain during vaccine manufacturing are thought to have contributed to low vaccine effectiveness estimates for H3N2 in other influenza seasons^26,27^.

Lower vaccine effectiveness estimates have been observed for H3N2 than for other strains since 2009, even during seasons when the selected vaccine strain appeared to be well matched to circulating strains^27^. Differences in HA glycosylation between the vaccine strains and circulating strains are thought to have contributed to this reduced vaccine effectiveness^26^. During the 2014–2015 influenza season, a clade 3 C.2a H3N2 strain possessing a new predicted HA glycosylation site emerged^26^. For the 2016–2017 season, the influenza vaccine was updated to include a clade 3 C.2a H3N2 strain (A/Colorado/15/2014) containing the new glycosylation site^26^. However, this particular glycosylation site was absent in the egg-adapted virus. Consequently, antibodies induced in humans, and in ferrets, poorly neutralised the glycosylated clade 3 C.2a H3N2 strain^26^. Contrary to the egg-derived vaccines and as expected, rHA containing the new glycosylation site induced optimal levels of antibodies that efficiently recognised the glycosylated clade 3 C.2a H3N2 virus^26^. The chances of introducing deleterious mutations through the adaptation of seed virus during vaccine manufacturing today are low due to the stringent quality control of the working seed virus. Indeed, as per current regulatory requirements, seed viruses must be confirmed for both genetic and antigenic match with their originating wild-type virus before they can be used for vaccine production. Nonetheless, the time it takes to generate appropriate seeds could hinder the timely availability of the vaccines.

Recombinant DNA technology avoids the risk of the virus acquiring egg- or cell-adapted mutations during the manufacturing process as it does not use ‘live’ influenza virus. Instead, DNA coding for HA is cloned from a reference virus published in the G*lobal Initiative on Sharing All Influenza Data* (GISAID) database and is confirmed for fidelity at the working virus bank level^28^. As such, the primary amino-acid sequence of the rHA produced using baculovirus or other recombinant expression system is identical to the HA from the wild-type virus isolate selected for seasonal influenza vaccine production. Thus, the risk of antigenic mismatch of RIV4, or other rHA vaccines in development, with the wildtype influenza strain selected for vaccines is eliminated.

## Recombinant HA expression systems

Both prokaryotic and eukaryotic expression systems have been used for the manufacture of rHA vaccine antigens. The first candidate recombinant influenza vaccines to be successfully manufactured using an *Escherichia coli* fermentation system involved expression of the globular head domain of the HA protein genetically fused with the Toll-like receptor 5 agonist, *Salmonella typhimurium* flagellin type 2^29^. The resultant vaccines elicited strong and protective antibody responses in mouse models^29^. In Phase 1 clinical evaluation, a prototypic quadrivalent vaccine developed using this *E. coli* platform elicited immune responses in healthy adults with favourable tolerability^30^. The *E. coli* expression system has been shown to generate a high yields of rHA (200 mg/L of purified HA protein) using a minimal number of bioreactors^31^. The authors projected that the strategy could yield up to half a billion doses of vaccine per month in a medium-scale pharmaceutical production facility^31^. This approach will likely shorten the entire vaccine manufacturing process^32^. However, *E. coli*-expressed rHA proteins can be subject to mis-folding, contain impurities (e.g., host-cell proteins), and do not undergo glycosylation^33^. They therefore need extensive processing to attain desired purity and to fold to their native conformation^31^. The resulting processed proteins are less immunogenic than egg-derived antigens, with around a 10-fold greater quantity needed to generate protective immunity in animal models^31^. These inherent complications have prevented large scale manufacturing and eventual commercialisation of *E. coli*-expressed influenza vaccines.

Vaccines containing plant-derived rHA either in soluble form or in virus-like particles (VLPs) have been shown to be safe and immunogenic in humans^34–37^. A plant-derived recombinant quadrivalent VLP (QVLP) at 30 µg dose per strain was found to be non-inferior in terms of vaccine efficacy against respiratory illness and influenza-like illness to a quadrivalent inactivated influenza vaccine (QIV; Fluarix Quadrivalent, GlaxoSmithKline) given at 15 µg dose/strain in adults aged 18 to 64 years^37^. However, inconsistency in the expression levels of target proteins, due to nonspecific integration of transgene(s) into the host genome, has been a major challenge with plant-based expression systems^34^. The unpredictable yield could adversely impact timely vaccine production, which typically involves updating at least one HA component of the vaccine to reflect antigenic change in the circulating influenza viruses each season. A unique positive attribute of plant-derived rHA is that it can stimulate innate immunity that predominantly facilitates Type 1 pro-inflammatory cellular immune responses, potentially as a direct effect of the plant-origin lipids/glycolipids in the vaccine formulation^38^. These stimulatory components in a plant-derived vaccine may need to be controlled and kept at a constant level, for commercialisation, to avoid potential severe adverse events caused by enhanced immune responses in vaccinees.

Several groups have explored the use of adenovirus (AdV), primarily as a replication-defective vector, for gene delivery and transgene expression of rHA in the host cells. AdV vectors induce both cell-mediated and humoral immunity against the expressed protein, providing robust protection against the targeted disease^39–41^. However, pre-existing or acquired immunity against adenovirus can hamper vaccine effectiveness by neutralising the vaccine vector and clearing the vector-infected cells. While the use of non-human adenovirus vectors could potentially overcome this issue, AdV vector immunity developed through repeated immunisation (for example during repeated annual influenza vaccinations) can dampen the immune response against the vaccine antigens^39,40^.

**Fig. 1:**
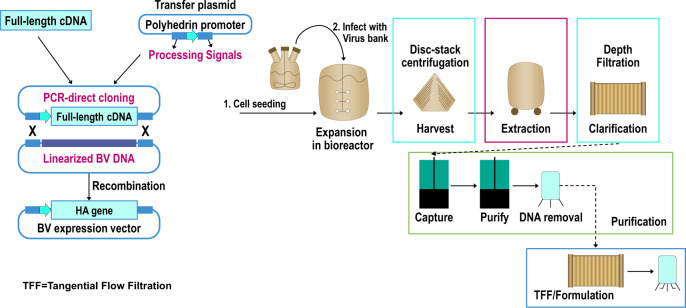
Generation of rHA using the baculovirus-insect cell expression system for the manufacture of RIV4. BV baculovirus. Figure adapted from Cox, M. M. & Hashimoto, Y (2011). A fast track influenza virus vaccine produced in insect cells. *J Invertebr Pathol* 107 Suppl, S31-41 ^©^ 2011 Elsevier Inc, with permission from Elsevier^23^.

RIV4 is one of only two available influenza vaccines that are egg-free. Like other recombinant vaccines, expression of HA in insect cells does not depend on the generation of reassortant candidate vaccine viruses selected for robust growth in embryonated eggs or mammalian cell-lines. Instead, HA genetic sequences from the wild-type strains selected for inclusion in the vaccine are inserted into the BEVS, from which high yields of rHA are harvested and purified (Fig. 1). This process requires less than half the time (2–3 months) required for the manufacture of conventional influenza vaccines^42,43^. This is a critical advantage for the timely supply of influenza vaccine for both seasonal epidemics and in pandemic situations. VLPs have been produced successfully by integrating HA, capsid protein (M1) and neuraminidase (NA) expressed in insect cells^44–46^. Although presenting proteins in VLP structures enhanced their immunogenicity, it compromised the purity of the vaccines considerably, as VLP structures also integrated both baculovirus and Sf9 cell proteins. Elimination of these unwanted proteins from the vaccine required extensive disassembly and reassembly processing of purified VLPs, which is a constraint for the commercial scale manufacturing of vaccines annually. It also raises the cost and the time it takes to bring the vaccines to the market. The yield and the cost of various manufacturing processes and technologies are critical elements to ensure adequate supply of vaccine at an affordable cost, especially in a pandemic situation. This aspect of the vaccine manufacturing has been discussed exhaustively in a report published by the Program for Appropriate Technology in Health (PATH) and is not covered here^47^.

**Fig. 2:**
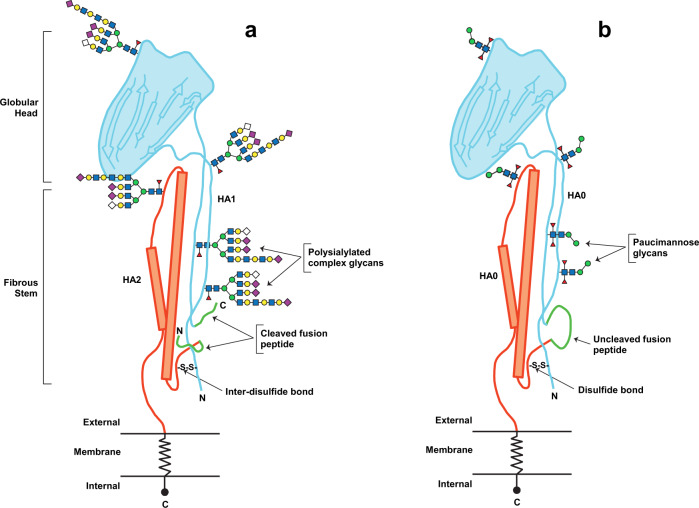
Structural features of native HA expressed on influenza virus and rHA produced in insect cells using the baculovirus expression vector system. **a** HA protein (shown as monomer) extracted from influenza virus is a heterodimer comprising HA1 (turquoise) and HA2 (orange) linked though an inter-disulfide (S–S) bond and contains complex-type sialylated N-linked glycans. Cleaved fusion peptides (green) and complex glycans^74^ eliminate and mask unique epitopes on HA respectively. **b** Recombinant HA protein (shown as monomer) expressed in insect cells as a single precursor polypeptide (HA0) with amino acid sequence identical to wildtype HA sequence and contains unsialylated paucimannosidic N-linked glycans^74^. HA0 is coloured in turquoise and orange to show HA1 and HA2 segments combined as a single polypeptide. Uncleaved fusion-peptide loop (green) and simpler glycans preserve and expose unique epitopes on rHA.

Insect cell-derived rHA (RIV4) has been studied extensively by various groups in both pre-clinical models and humans. The rHA contained in RIV4 differs from that expressed in other systems in terms of specific structural features, the nature of the source material and the manufacturing process, which have an impact on certain aspects of vaccine safety and efficacy (Fig. 2). Based on these unique features, RIV4 received ‘product exclusivity’ protection from FDA, a ‘new active substance’ designation from EMA Committee for Medicinal Products for Human Use (CHMP)^48^ and an ‘innovative drug’ designation from Health Canada^49^. The use of this technology, together with the molecular characterisation of the product, should facilitate continued evolution of influenza vaccines with improved effectiveness and their timely availability to the public.

## The tertiary structure of recombinant HA produced in insect cells can influence vaccine immunogenicity

Influenza HA is synthesised as a single precursor polypeptide (HA0), which is generally cleaved into two polypeptides (HA1 and HA2) extracellularly by trypsin-like serine proteases, furin and other subtilisin family endoproteases after newly synthesised virions are released from infected cells^50,51^. HA cleavage is essential for the infectivity of influenza virus. Thus, expression of these proteases exclusively in the respiratory tract is responsible for influenza virus tropism to epithelial cells of the upper and lower respiratory tract. However, HA from influenza subtypes H5 and H7 contains multi-basic cleavage sites that are cleaved by ubiquitously expressed furin in the trans-Golgi network, making these strains highly virulent^50,52,53^. The HA1 and HA2 polypeptides remain covalently linked by a disulfide bond to form heterodimers (Fig. 2). These molecules, either uncleaved HA0 or cleaved HA1–HA2 heterodimers, are expressed on the viral membrane surface as trimers (HA ‘rosettes’). The conversion of HA0 to HA1–HA2 heterodimers leads to conformational changes that render the molecule fusion-competent, thus enabling the virus to infect a new host cell^52,54^. In inactivated (split and subunit) vaccines, the HA molecules derived from live influenza viruses are present predominantly as HA1–HA2 heterodimers. By contrast, the rHA included in RIV4 is designed to yield predominantly as a single HA0 polypeptide (Fig. 2). A previous biochemical study (using SDS-PAGE under reducing conditions) clearly demonstrated the predominance of HA0 in recombinant HA H7 subunit complexes, produced using a BEVS, with no protein bands indicative of HA1 or HA2 molecules^55^. Poor processing of HA0 to HA1 and HA2 polypeptides in Sf9 cells has been shown to be due to insufficient levels of furin-like proteases in the cells^56^.

The conformational differences between pre-fusion and post-fusion states of HA and the process of exposure of the fusion peptide have been well described^54^. Webster et al.^57^ demonstrated, using monoclonal antibodies, that neutralising epitopes present in the pre-fusion HA0 molecule were lost upon conversion to the post-fusion HA1–HA2 heterodimer at an acidic pH^57^. Similarly, several broadly neutralising antibodies directed against the highly conserved HA stem region have been shown to block HA maturation and fusion^58^. Some of these antibodies are likely to bind near the uncleaved fusion peptide that protrudes at the surface of the HA0 rosettes. Turner et al.^59^ identified a monoclonal antibody that binds to an epitope on HA molecules that are partly and transiently exposed in the pre-fusion conformation^59^. Structural analysis of the antigen-antibody complex revealed a potential dynamic state where HA undergoes structural fluctuations in its pre-fusion state^59^. A recent study by Khurana et al.^60^ further demonstrated, using surface plasmon resonance technology, that the observed broader specificity of antibodies induced by RIV4 may be linked to the presence of unique epitopes on HA0^60^. Additionally, several groups have isolated, using HA0 as the immunogen or from a phage display library, protective antibodies specific to epitopes in the fusion loop region present only in HA0^61,62^. These antibodies exhibited unprecedented breadth and potency and neutralised a diverse panel of representative viruses in group 1 and group 2 influenza A, blocked protease cleavage of HA0 and locked HA in the pre-fusion state. Thus, these antibodies make the virus non-infectious by inhibiting the pH-induced conformation change and the HA-mediated membrane fusion that are essential for the virus infectivity. HA molecules presented on influenza virions are predominantly in the pre-fusion state. Once the virus binds to a cell or enters the acidic endosome of infected cells, the fusion loop is cleaved to yield HA1 and HA2, and the hydrophobic fusion peptide in the N-terminus of HA2 is sequestered away from membranes in a pocket, limiting its exposure^52,63^. Nonetheless, it would be of interest to examine the immunogenicity of released HA fusion peptides in the post-fusion state and their potential role in protection, as these are present in conventional influenza vaccines. Together, these critical findings clearly demonstrate structural differences between HA pre-fusion and post-fusion states, and the presence of unique neutralising epitopes in the pre-fusion HA0 molecules, which are present in RIV4 (Fig. 2).

Structural differences in the HA polypeptides and rosettes between recombinant and split vaccines have been shown to result in differences, both qualitative and quantitative, in the immune response to vaccines in humans and animals. Portnoff et al.^64^ demonstrated that recombinant HA antigens (specifically for the H3 strain) produced using BEVS (as used for RIV4), induced significantly higher levels of broadly cross-reactive antibodies against highly conserved regions of the HA head and stem domains than egg-derived split vaccines^64^. Recently Richards et al.^65^ examined CD4 T-cell and antibody responses in healthy adults who received egg-derived split vaccine, cell-derived split vaccine or RIV4 for three successive influenza seasons (2015–2016, 2016–2017, and 2017–2018)^65^. RIV4 elicited the most robust responses, with significantly higher T-cell and antibody levels than the other two vaccines. Authors postulated that simpler glycosylation of rHA and absence of other influenza viral proteins in RIV4 contributed to the observed robust immune response for RIV4 and emphasised the relevance of these features in determining vaccine efficacy and long-term immunity^65^.

## The homogeneity of recombinant HA antigens in RIV4 may improve the safety profile over conventional influenza vaccines

As described above, HA is expressed on the viral surface as a HA trimer (rosette). When these are extracted from influenza viruses, they form clusters of varying sizes. This has been observed in conventional influenza virus-derived vaccines, with estimates of 18 to 1100 trimers per cluster^55,66,67^. Two distinct populations of cluster have been observed; the majority have an average diameter of 150 nm, while the remainder are larger (average diameter, 5500 nm)^66^. However, the rosette clusters in RIV4 are uniform in size and presentation, containing around six to eight HA trimers per cluster at an average diameter of approximately 30–40 nm^55,67^. The elution of rHA from RIV4 drug substance as a single peak in size-exclusion high-performance liquid chromatography is monitored for the release and the stability of RIV4.

Further characterisation by cryo-electron microscopy has revealed that the HA rosette clusters in RIV4 are uniformly starfish-shaped; whereas in the split vaccine they are mostly non-symmetrical and agglomerated into huge structures, resulting in both starfish- and peanut-shaped heterogeneous particles^55^. Additional electron microscopy analysis of split vaccine clusters showed a highly heterogeneous mixture containing different types of viral particles containing HA and NA as well as split viral membrane folded in various shapes, slightly disrupted virions, and whole virions^66^.

In Canada between 2000 and 2004, an unusual number of cases of a syndrome termed as oculo-respiratory syndrome (ORS) were reported following immunisation with the inactivated influenza split-virus vaccine^68^. Detailed analysis revealed that ORS, induced within 2 to 24 h of vaccination, was suspected to be due to the presence of micro-aggregates of unsplit virions in the conventional egg-derived influenza vaccines^69^. High levels of aggregate content in the split vaccine are believed to have induced a Type-2 polarised immune response resulting in ORS based on study results from a mouse model^70^. Although extremely rare, this is unlikely to occur with RIV4 that contains HA rosettes of small and uniform size.

## The N-linked glycan structure of recombinant HA produced in insect cells differs significantly from HA in other influenza vaccines

Influenza HA has a variable number of N-linked glycosylation sites (depending on the virus strain and subtype) in the globular head region and the conserved stem region^71^. The glycosylation of HA has various functions, including regulation of the virus life-cycle and a role in disease pathogenesis^71^. During vaccine manufacture, the host cells used for the production of HA play a major role in determining HA N-glycan composition^72,73^. Glycoproteins expressed in mammalian cells typically have sialylated complex-type N-linked glycans, while those expressed in insect cells typically have simple unsialylated glycans (either truncated, paucimannosidic or oligomannosidic glycans)^74^. An et al.^72^ showed that egg-derived and mammalian (Madin-Darby canine kidney) cell-derived HA predominantly contained highly-branched complex or high-mannose glycans, whereas HA expressed in Sf9 insect cells had relatively small paucimannose glycans (Fig. 2)^72^.

The peptide sequences around glycosylation sites are highly conserved and, as such, antibodies directed against these regions could provide broader specificity. Antisera raised against simple monoglycosylated HA in mice were shown to improve the breadth and capacity of HA-neutralising antibodies to protect against lethal challenge with H5N1 compared to antisera raised against fully glycosylated HA^75^. Thus, elimination of parts of glycans that are not essential for HA structure may improve vaccine-induced protection. Subsequent studies showed that HA with simpler glycans induce more broadly protective antibodies with superior cross-clade protection compared to HA with more complex glycans^76–79^. The presence of simpler glycans appears to be equally efficient to that of chicken or mammalian cell-derived glycosylation in ensuring the proper folding of HA, and exposes conserved regions of the molecule for the induction of immunity with broader protection^12,71,76–80^.

In a study by Nachbagauer et al.^78^, RIV4 induced HA stem-specific neutralising antibodies directed against influenza subtypes H1, H3 and B haemagglutinin in an age-dependent manner in humans, with the highest titres observed in the elderly^78^. RIV4 also induces antibodies, in both humans and mice, that are specific to epitopes in the HA head region, at greater proportions than a traditional mammalian cell-derived subunit vaccine (Flucelvax^®^ [Trivalent])^77^. Higher magnitudes of haemagglutination inhibitory antibody response against HA1 have also been observed with RIV4 compared to egg- or mammalian cell-derived split vaccines (Flucelvax^®^ Tetra, Seqirus, and Fluzone^®^ quadrivalent SD, Sanofi Pasteur)^60^. These data warrant additional studies to verify whether rHA elicits a broader antibody repertoire than conventional vaccines and whether this underlies the cross-protection against antigenic drift variants previously observed in clinical trials^12,81^.

RIV4 has a unique ability to induce broadly cross-reactive antibody responses to antigenically drifted A/H3N2 viruses in humans. In a small study by Belongia et al.^82^, participants aged 65–74 years were immunised with RIV4, a high-dose split-virion inactivated trivalent influenza vaccine (Fluzone^®^ High Dose, Sanofi Pasteur; HD-IIV3) or adjuvanted-IIV3 (aIIV3)^82^. Participant sera were tested against four A/H3N2 viruses including a cell-propagated reference vaccine strain, two circulating viruses and an antigenically advanced virus with evidence of antigenic drift. The post-vaccination geometric mean fold rise against the two circulating viruses was twice as high for RIV4 as for HD-IIV3 or aIIV3. Post-vaccination titre against the antigenically drifted H3N2 were generally low and similar across all groups, however, receipt of RIV4 was strongly associated with seroconversion to this strain (*p* = 0.003). The investigators suggested that although the circulating A/H3N2 viruses were antigenically similar to the cell-grown vaccine reference virus, egg propagation of the vaccine strains had led to loss of a glycosylation site and impaired antibody response to circulating viruses, consistent with previous reports^26^.

In addition to the above referenced studies, in a recent study by Shinde et al.^83^ baculovirus-expressed rHA generated cross-protective responses against both circulating and drifted A/H3N2 strains, including in older adults who are at a higher risk for influenza and associated medical complications^83,84^. This unique characteristic of the recombinant vaccine is likely related to glycosylation of rHA in the insect cell line, leaving it uncleaved. The higher quantity and greater accessibility of the genetically conserved stem region of rHA produced in insect cells (resulting in smaller N-linked glycans) may contribute to cross-protection against mismatched influenza strains^12,75^. The study by Nachbagaeur et al.^78^ supports the hypothesis that a recombinant vaccine results in increased titre of broadly neutralising HA stem-reactive antibodies and that these immune responses increase with age^78^. This increase with age is possibly due to repeated exposure to divergent influenza viruses similar to the multiple A/H3N2 virus strains evaluated by Belongia et al.^82^. Therefore, vaccine constructs that preserve the highly conserved HA stem can protect against drifted viruses and thus may confer a greater breadth of protection against influenza.

## Recombinant HA antigens are not subjected to chemical modifications

In all conventional influenza vaccines, whether derived from eggs or mammalian cells, the antigens are exposed to inactivation agents such as formaldehyde or β-propiolactone (BPL). These inactivating agents cause numerous modifications to the antigenic epitopes on HA through cross-linking or formation of bis-alkylated groups^85–87^. These modifications often result in changes to protein folding, conformation, and stability^88^. Unlike conventional vaccines, RIV4 does not go through an inactivation step, thus preserving the native HA conformation of the wild-type virus, required for optimal protective immune response.

## Absence of egg or other influenza viral components in recombinant HA vaccine is likely to favour its safety profile

As most split and subunit vaccines are prepared from influenza viruses that are grown in embryonated chicken eggs, they contain egg proteins. Testing of 58 vaccine lots covering six different seasonal influenza vaccines produced by five different manufacturers showed that the median ovalbumin concentration was 350 ng/mL^89^. Moreover, another study of commercial influenza vaccine preparations detected other viral proteins such as nucleoprotein and matrix protein (confirmed by a chromatographic separation) that are not shown to be relevant for vaccine effectiveness, and viral RNA fragments (confirmed by activity assay)^90,91^. Such egg-derived or other influenza virus-derived proteins (as described above) and influenza viral RNA fragments are not present in recombinant vaccines.

Although uncommon, some individuals may be at increased risk of hypersensitivity reactions to component proteins such as ovalbumin and those who are sensitised may be at higher risk of clinical manifestations of allergic disease upon exposure^92^. Egg protein, viral RNA, and process impurities such as inactivating agents or hydrocortisone that are typically present in conventional vaccines are absent in RIV4 would eliminate potential adverse reactions, although rare, to these components in vulnerable individuals^93^.

## Conclusions

The evidence reviewed here demonstrates several advantages of the BEVS used in the manufacture of RIV4 over conventional influenza vaccines that rely on influenza virions propagated in egg or mammalian cells, split with detergents with or without further HA enrichment. The use of recombinant protein technology eliminates the risk of antigenic mismatch due to potential changes in primary HA structure through egg- or cell-adaptation. We also describe features of the rHA tertiary structure that are likely to be responsible for the generation of broad cross-reactive and protective antibodies, together with the direct or indirect evidence supporting this. The homogeneity of rHA rosettes and negligible process-related impurities are the hallmarks of RIV4. As rHA production bypasses the need for a viral inactivation step and avoids the use of eggs, related process-impurities such as inactivating agents or residual egg-protein, and thus potential adverse reactions to these impurities in vulnerable individuals are eliminated. This well-established and validated platform for vaccine manufacture could be extended to address other emerging infectious diseases where cross-protection against constantly evolving variants is critical, such as pandemic influenza and/or COVID-19.

## Data Availability

No data were generated for the review article.
